# Genetic Control of Rod Bipolar Cell Number in the Mouse Retina

**DOI:** 10.3389/fnins.2018.00285

**Published:** 2018-05-09

**Authors:** Amanda G. Kautzman, Patrick W. Keeley, Sarra Borhanian, Caroline R. Ackley, Benjamin E. Reese

**Affiliations:** ^1^Neuroscience Research Institute, University of California, Santa Barbara, Santa Barbara, CA, United States; ^2^Department of Psychological and Brain Sciences, University of California, Santa Barbara, Santa Barbara, CA, United States; ^3^Department of Cellular, Molecular and Developmental Biology, University of California, Santa Barbara, Santa Barbara, CA, United States

**Keywords:** recombinant inbred strain, quantitative trait locus, structural variant, electroporation, luciferase assay, *Ggct*

## Abstract

Genetic variants modulate the numbers of various retinal cell types in mice. For instance, there is minimal variation in the number of rod bipolar cells (RBCs) in two inbred strains of mice (A/J and C57BL/6J), yet their F1 offspring contain significantly more RBCs than either parental strain. To investigate the genetic source of this variation, we mapped the variation in the number of RBCs across 24 genetically distinct recombinant inbred (RI) strains (the AXB/BXA strain-set), seeking to identify quantitative trait loci (QTL). We then sought to identify candidate genes and potential casual variants at those genomic loci. Variation in RBC number mapped to three genomic loci, each modulating cell number in excess of one-third of the range observed across the RI strains. At each of these loci, we identified candidate genes containing variants that might alter gene function or expression. The latter genes were also analyzed using a transcriptome database, revealing a subset for which expression correlated with variation in RBC number. Using an electroporation strategy, we demonstrate that early postnatal expression of one of them, *Ggct (gamma-glutamyl cyclotransferase)*, modulates bipolar cell number. We identify candidate regulatory variants for this gene, finding a large structural variant (SV) in the putative promoter that reduces expression using a luciferase assay. This SV reducing *Ggct* expression *in vitro* is likely the causal variant within the gene associated with the variation in *Ggct* expression *in vivo*, implicating it as a quantitative trait variant (QTV) participating in the control of RBC number.

## Introduction

The nervous systems of different laboratory mouse strains show conspicuous phenotypic variation, and often that variation can be traced to causal genetic variants. Some of those variants are disease-causing mutations (Chang et al., 2013) or affect susceptibility genes (Libby et al., 2005), while others modulate quantitative traits, for instance, the size of a brain region (Airey et al., 2001; Williams et al., 2001; Peirce et al., 2003; Mozhui et al., 2007), or the number of neurons in a structure (Seecharan et al., 2003). Different mouse strains have been shown to contain large differences in the number of particular types of neurons within the retina (Williams et al., 1998), in some cases exhibiting a two-fold or greater variation (Keeley et al., 2014), and much of that variation has been mapped to genomic loci where such causal variants must be present. In some of those cases, candidate genes containing putative causal variants have been identified, and gene-knockout mice have confirmed an effect upon cell number directly (Whitney et al., 2009, 2011a).

We recently observed minimal variation in the number of rod bipolar cells (RBCs) between the retinas of two different inbred laboratory strains of mice, the C57BL/6J strain, and the A/J strain. Yet when crossed to generate reciprocal F1 offspring, those F1 strains contain substantially more RBCs than the parental strains, suggesting the presence of variant genes that contribute to the establishment of this trait. A number of transcription factors have been shown to modulate the determination, differentiation, or survival of bipolar cells in the mouse retina, some of which modulate exclusively RBCs (Burmeister et al., 1996; Tomita et al., 2000; Bramblett et al., 2004; Chow et al., 2004; Feng et al., 2006; Elshatory et al., 2007; Shi et al., 2011, 2012; Star et al., 2012), raising the possibility that variants within these genes might contribute to such strain differences. Alternatively, other novel genes might contain causal variants discriminating the two parental strains. The present study has examined a panel of recombinant inbred (RI) strains derived from these same parental strains (the AXB/BXA strain set), seeking to map quantitative trait loci (QTL) for RBC number. We describe QTL on three chromosomes (Chrs 4, 6, and 8), and in turn interrogate those loci, identifying candidate genes based on the presence of putative coding or regularity variants discriminating the parental genomes. We were particularly interested in a number of genes for which their own expression correlated with the variation in RBC number across the RI strains, and for which that variation in expression mapped to the genomic location of the genes themselves (i.e., a *cis*-expression QTL, or *cis*-eQTL), suggesting that transcript abundance participates in the modulation of cell number. We tested this directly by electroporating expression-plasmids for one of these genes, *Ggct*, finding that its expression reduced the proportion of retinal bipolar cells. Finally, we identified a structural variant (SV) upstream of the transcriptional start site (TSS) in the A/J genome and confirmed its suppressive effect on gene expression via luciferase assay.

## Materials and methods

### Tissue collection and immunofluorescence

C57BL/6J (hereafter B6/J) and A/J mice, their reciprocal F1 offspring (AB6F1 and B6AF1), and 24 genetically distinct RI strains from the AXB/BXA strain set were obtained from the Jackson Laboratory or were bred at UCSB in the Animal Resource Center. Mice 4–8 weeks of age were given an intraperitoneal injection of 120 mg/kg sodium pentobarbital (Euthasol; Virbac Animal Health) and were then perfused intracardially with ~2 ml of 0.9% saline followed by ~50 ml of 4% paraformaldehyde in 0.1 M sodium phosphate buffer (pH 7.2 at 20°C). Eyes were enucleated and immersed in 4% paraformaldehyde for 15 min at room temperature for additional fixation of the tissue. Electroporated retinas were fixed by immersion fixation in 4% paraformaldehyde for 30 min. These procedures were conducted under authorization by the UCSB Institutional Animal Care and Use Committee and in accord with the NIH *Guide for the Care and Use of Laboratory Animals*.

Retinas were dissected from the eyes taking care to maintain the entirety of the retina, and four radially-oriented relieving cuts (extending half-way to the optic nerve head) were made to enable the retina to lie flat. The vitreous was gently brushed from the inner surface of the retina, and the wholemount then rinsed in sodium phosphate buffer and incubated for 3 h in a blocking solution containing 5% normal donkey serum. For RBC counts across parental, F1, and RI strains, one retina from each mouse was then incubated in a polyclonal rabbit antibody to protein kinase Cα*βγ* (PKC, 1:10,000, Cambio, #CA1042) for 3 days, and incubated in donkey anti-mouse secondary antibody conjugated to Cy3 (1:200; Jackson ImmunoResearch Labs, #715-165-150) overnight at 4°C. Electroporated retinas were incubated in a polyclonal rabbit antibody to green fluorescent protein (GFP, 1:1,000, Molecular Probes, #A21311). Developmental and adult B6/J retinas were embedded in 5% agarose and sectioned perpendicular to the plane of the retina into 150 μm radial sections on a Vibratome. Sections were incubated in a polyclonal rabbit antibody to Ggct (Thermo Fisher Scientific, 1:100, #PA554263) for 3 days, and incubated in donkey anti-rabbit secondary antibodies conjugated to AlexaFluor488 (1:200; Jackson ImmunoResearch Labs, #711-545-152) overnight at 4°C. (Control sections, in which the primary antibody had been omitted, showed the absence of any labeling). All solutions were prepared in 0.1 M phosphate buffer (PB) with 1% Triton-X100 (Sigma), and all steps were carried out at 4°C with agitation.

### Quantification of cell number

**Figure 1:**
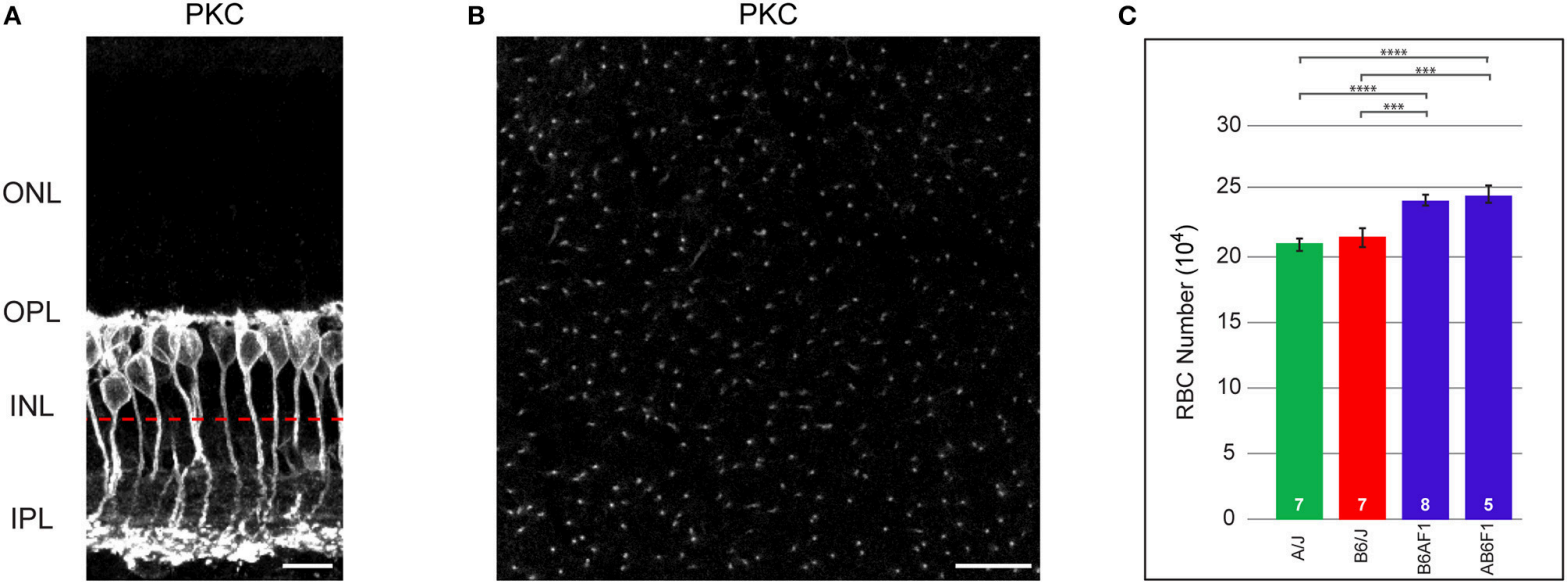
**(A)** RBCs, labeled with antibodies to PKC, are closely packed at the outer margin of the INL. RBC axons course through the INL to reach the IPL, where they terminate in the inner-most ON stratum. **(B)** When imaged in wholemount preparations, individual axons are reliably discriminated. The approximate focal plane in **(B)** is indicated by the dashed red line in **(A)**. **(C)** Total number of RBCs in the parental B6/J and A/J strains, and in their reciprocal F1 offspring, B6AF1 and AB6F1. Each parental strain differs significantly from each F1 strain, but the two parental strains and the two F1 strains do not differ significantly from one another. Scale bar: **(A)** = 10 μm; **(B)** = 25 μm. ^***^p < 0.001; ^****^p < 0.0001.

**Figure 2:**
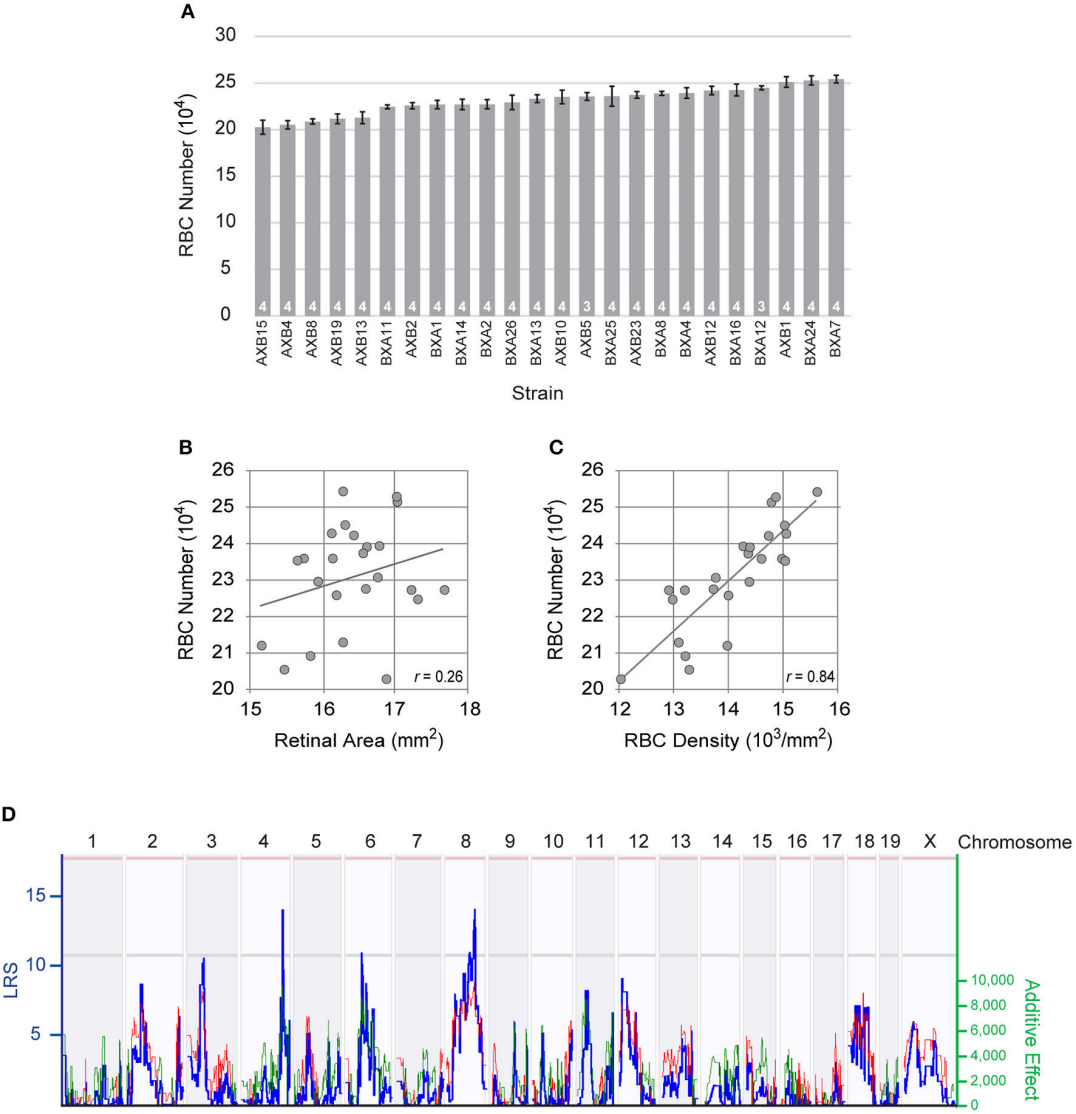
**(A)** The total number of RBCs showed large variation across the RI strains of the AXB/BXA strain set, while evidencing only modest within-strain variation. *n* = the number of retinas sampled per strain. **(B)** Retinal area varied slightly across the RI strains, but did not correlate significantly with RBC number. **(C)** Rather, the density of RBCs correlated significantly with RBC density. The Pearson correlation coefficient (*r*) is indicated. **(D)** Variation in RBC number mapped three large-effect quantitative trait loci (QTL) on Chrs 4, 6, and 8, where *A* alleles at the former loci and *B* alleles at the latter locus correlated with an increase in RBC number. The blue trace plots the likelihood ratio statistic (LRS), indicated on the left Y-axis. Suggestive and significant thresholds for the LRS defined by 1,000 permutation tests of the strain data are indicated by the gray and pink horizontal lines, respectively. The right Y-axis plots the additive effect of the contribution of a single allele upon trait values. The effect of two alleles at each of these three loci modulated the trait by 19,294 cells, 17,535 cells, and 18,413 cells, respectively. Note as well other lesser loci present across the genome, including those on Chrs 3 and 12.

Immuno-labeled retinas were mounted whole under a coverslip in 0.1 M sodium phosphate buffer with the ganglion cell layer oriented facing the coverglass and examined using an Olympus BH2 fluorescence microscope coupled via a Sony video camera to a computer. Four fields, each positioned at a mid-location between the retinal circumference and optic nerve head, were sampled, one in each quadrant of the retina, the areal size of each sampling field being 0.016 mm^2^. Every PKC immuno-positive axon coursing through the inner nuclear layer (INL) and inner plexiform layer (IPL) within the sampling field was counted, and the area of each retinal wholemount was measured using Bioquant Nova Prime software (R&M Biometrics). Retinal area was multiplied by the average density of RBCs across the four fields in order to estimate total RBC number. A minimum of four mice were sampled in all strains but one (BXA12), the n being indicated in each bar of the histograms in Figures 1, 2, showing the mean total RBC number and standard error per strain. Typically, three strains of mice were processed at the same time, with all mice individually coded and randomly sorted, so that all counting was conducted blind to strain, with the same individual analyzing all 122 mice (see Keeley et al., 2017, for full procedural details). Individual fields for illustration were imaged using an Olympus Fluoview 1000 laser scanning confocal microscope with a 40 × objective, in which images were collected at 1 μm intervals through the INL and prepared as Z-stack projections.

### QTL mapping and interval analysis

Simple interval mapping was conducted with the mapping module at GeneNetwork (www.genenetwork.org). The original phenotype data (RBC total number) have been entered into the AXB/BXA Phenotypes database in GeneNetwork as accession record ID #10202, and a preliminary report of the mapping exercise has been published, including the strain data in an on-line appendix (Keeley et al., 2014). Permutation testing of the RI strain data was conducted to determine the probability of achieving a likelihood ratio statistic (or LRS, being an index of the strength of the linkage between genotype and phenotype) by chance. Thresholds for suggestive (*p* = 0.63) and significant (*p* = 0.05) LRS scores are indicated by the horizontal lines in Figures 2D, **4B**. The megabase (Mb) position values described in this study refer to the Mouse Genome Assembly of 2011 (GRCm38/mm10).

Variants within a genomic region were found using the Sanger Institute Mouse Genomes Project database (Keane et al., 2011; Yalcin et al., 2011), and divided into two categories: coding and regulatory variants. For the initial screening of genes at a QTL we consider variants in all potential transcripts. Coding variants consisted of single nucleotide polymorphisms (SNPs) or small insertions/deletions (InDels) that fell within the coding region of a gene, and were predicted to cause frameshift mutations, STOP mutations, missense mutations, or inframe InDels. Missense mutations were further run through the SIFT database to predict whether the amino acid substitution will be deleterious to protein function (Ng and Henikoff, 2003). Potential regulatory variants were defined as any variant that fell within 2 kb upstream of the TSS, which we have defined as the size of the putative promoter region, in addition to variants in 5′ or 3′ untranslated regions (UTR), downstream regions (2 kb), or introns. To determine if any potential regulatory variants created novel transcription factor binding sites, A/J and B6/J sequences were aligned in zPicture (zpicture.dcode.org) and then compared with rVista (rvista.dcode.org), using the TRANSFAC professional V10.2 library of vertebrate transcription factor binding site matrices. Only high-specificity matrices with a predefined matrix similarity cutoff of 0.9 were used to restrict the output to only the most likely binding sites.

The Mouse Retina SAGE library (Blackshaw et al., 2004) and C57BL/6J retinal microarray expression analysis (Freeman et al., 2011) were used to determine the expression of those genes housing parental genetic variants in the developing and adult retina, respectively. All genes that met these two criteria where investigated in further detail for known functions described in the literature. A microarray database examining adult whole eye mRNA from this same set of RI strains was also consulted (Whitney et al., 2011b), to identify from this list any genes exhibiting variation in their expression levels that correlated to the variation in RBC number, and for which that variation in expression mapped a *cis*-eQTL.

### Quantitative PCR

For real-time RT-PCR (qPCR) experiments, a minimum of 6 retinas were collected from three separate litters of A/J and B6/J animals at P1, P5, and P10 in RNase-free conditions. RNA was extracted using an RNeasy Plus Mini Kit (Qiagen, Hilden, Germany) and single-stranded cDNA was synthesized using the iScript cDNA synthesis kit (Bio-Rad, Hercules, CA). Expression levels of *Ggct* were measured using the CFX96 Touch Real-Time PCR detection system in conjunction with the SsoAdvanced Universal SYBR Green Supermix (Bio-Rad, Hercules, CA), using the recommended product protocol and empirically determined annealing temperatures for each primer set. Each primer set was run on a separate plate and all samples were run in triplicate, using the median rather than mean values, to minimize the effect of rare outliers. RNA abundance was corrected for product size, primer melting temperature, and primer efficiency, which was determined by creating a standard curve for each primer pair. Levels of *Ggct* expression were normalized to the geometric mean of three control genes, Glyceraldehyde 3-phosphate dehydrogenase (*Gapdh*), TATA-binding protein (*Tbp*), and β-2 microglobulin (*B2M*). The primer sequence, product size, annealing temperature, and calculated efficiency for each primer set are provided in Supplementary Table 1. PCR amplification of genomic A/J and B6/J DNA, extracted from adult tail tissue, was carried out using the following primers, listed 5′-3′ (*Ggct* forward: GAGAAGAGAACCATATTGCCATGCC; *Ggct* reverse: CTGTGACCTGATGCTCCTGACG).

### Electroporation

*Ggct* cDNA (*Ggct* #: 30286931; GE Healthcare Dharmacon, Lafayette, CO) was cloned into the multiple cloning site of a pCAGIG vector (Addgene, Cambridge, MA; # 11159), creating a vector such that the ubiquitous CAG promoter drives the transcription of the *Ggct* coding sequence followed by an IRES-*EGFP* cassette. The pCAGIG plasmid alone, including the downstream IRES-*GFP* sequence, was used as a control vector. Experimental conditions were adapted from Matsuda and Cepko (2004) and de Melo and Blackshaw (2011) with adjustments made to optimize transfection efficiency, conducted under UCSB IACUC authorization. P2 (the day after birth) pups were anesthetized on ice and, following cessation of movement, the presumptive eyelid was severed with a 27.5 gauge needle. A small hole was made in the sclera of one eye and a 32 gauge Hamilton syringe was used to inject 0.6–0.8 μl of plasmid DNA (1.5 μg/μl) into the sub-retinal space, after which five 50 ms square wave pulses of 80 V at 950 ms intervals were delivered using tweezer electrodes (BTX ECM 830, Holliston, MA). Pups recovered in a dish floating in a 42°C water bath and were returned to the dam upon regaining ambulation. Eyes were harvested at P21 as previously described, were incubated in rabbit anti-GFP antibodies and then scanned for GFP-positive regions indicating effective electroporation. Those retinas exhibiting GFP-positive regions were sectioned and all sections exhibiting GFP immunofluorescence were quantified. We documented the relative frequency of cells in three retinal sub-divisions: the ONL (photoreceptors), outer INL (bipolar cells), and inner INL (amacrine cells). GFP-positive Müller cells, residing in the middle of the INL, were binned into the outer INL or inner INL categories based upon subjective determination of the side in which more than 50% of the soma was positioned. All counts were done blind to condition.

### Luciferase assay

The following PCR primers were used to amplify *Ggct* promoter sequences from A/J and B6/J tail tissue, including adapters and restriction enzyme sequences listed 5′-3′ (*Ggct* forward: TAAGCAGAGCTCGAACTCACAGAGAACTGCCTGCCTC; *Ggct* reverse: TAAGCAAAGCTTCAGAGAAGCCGGACTAGCGCTG). The destination vector, also used as a control, was the pGL3-basic vector (Promega, Madison, WI; #E175A), to test the ability of the *Ggct* promoter sequences to drive the transcription of luciferase.

Plasmids were transiently transfected into HEK293T cells. The cells were grown in high glucose (4.5 g/L) DMEM media plus 10% FBS, with penicillin (100 U/mL) and streptomycin (100 μg/mL), in a T75 tissue culture flask. When the cells reached 80% confluence they were passaged, and plated in 12-well tissue culture plates at 1.5 × 10^5^ cells per well. Media was changed 24 h after plating, and cells were transfected 48 h after plating, with the proprietary transfection reagent TurboFect (Thermo Scientific, Carlsbad, CA; #R0531). Four hundred nanograms of experimental or control plasmid was transfected into each well, along with 20 ng of a plasmid encoding β-galactosidase (β-gal), and the transfectant was removed 13–16 h later. Cell lysates were collected 24 h from the start of transfection using 100 μl per well of cold 1X Passive Lysis Buffer (Promega, Madison, WI; #E1941).

Cell lysates were assayed for luciferase and β-gal activity on a Perkin-Elmer plate reader. Three biological replicate wells were assayed and averaged, each assayed twice to serve as a technical replicate. Luciferase units were corrected by subtraction of untransfected well units, and then were normalized using β-gal. The above experiment was replicated twice, with each of the three independent *Ggct* experiments yielding comparable (and significant) differences in luciferase expression, although the magnitude of the change exhibited high variability between experiments.

### Statistics

A one-way ANOVA was used to determine if there was an effect of strain when comparing RBC number in the parental and F1 strains, while Tukey's *post-hoc* tests were conducted to identify significant differences between individual groups. A two-way ANOVA was used to determine if transcript abundance for candidate genes exhibited main effects for strain or developmental age, or an interaction. A Student's one-tailed *t*-test was used to compare *Ggct* vs. control plasmid electroporation results. A one-way ANOVA was used to determine if there was an effect of promoter sequence when comparing Luciferase expression, while Tukey's *post-hoc* tests were conducted to identify significant differences between conditions. Pearson's correlation coefficient *r* was calculated to assess the relationship between total RBC number and retinal area or RBC density, and between transcript levels and RBC number. A *p*-value < 0.05 was used to determine statistical significance.

## Results

### Rod bipolar cell number shows conspicuous variation across RI strains

RBC somata are positioned at the outer margin of the INL (Figure 1A). They extend an apically directed dendritic stalk that gives rise to numerous branches within the outer plexiform layer (OPL), each of which terminates as a fine bulb invaginating into single rod photoreceptor spherules. From the basal end of the cell, a radial process courses through the remainder of the INL and into the IPL, terminating as a few large bulbous presynaptic terminals in the innermost part of the IPL. As in other species, these cells in mouse retina are readily labeled using antibodies to PKC, revealing all portions of the neuron for the entire population (Keeley and Reese, 2010). In wholemount preparations, they can be quantified either by counting their somata, or by counting their axons as they course basally toward the inner reaches of the IPL (Figure 1B). Such counts reveal nearly comparable numbers for the B6/J and A/J strains of mice, being 213,873 ± 6,595 cells and 209,009 ± 4,707 cells, respectively (red and green bars in Figure 1C). Their reciprocal offspring, being the AB6F1/J and B6AF1/J mice (where the female is denoted before the male parentage in each case), by contrast, had substantially greater numbers, with 244,523 ± 6,286 cells and 240,957 ± 3,425 cells, respectively (blue bars in Figure 1C). A one-way ANOVA comparing these four strains confirmed a significant effect of strain (*p* = 6.4 × 10^−5^), and Tukey's *post-hoc* tests showed that each F1 strain was different from each parental strain (A/J:AB6F1, *p* = 9.0 × 10^−4^; A/J:B6AF1, *p* = 7.4 × 10^−4^; B6/J:AB6F1, *p* = 4.0 × 10^−3^; B6/J:B6AF1, *p* = 4.0 × 10^−3^), but that there were no significant differences between the parental strains nor between the two F1 strains. This increase in the total number of RBCs observed in the F1 strains, relative to the parental strains, suggests the presence of countervailing variant genes that result in comparable RBC numbers between the parental strains.

To dissect the genetic basis of this variation, we examined the total number of RBCs in 24 distinct RI strains derived from these same parental strains (Figure 2A). Each RI strain has a unique combination of the two parental haplotypes, being homozygous at nearly all loci throughout the genome. The total number of RBCs across these strains varied more extensively than the difference observed in the parental and F1 strains, the lowest RI strain (AXB15) having only 202,415 ± 7,577 cells, while the highest RI strain (BXA7) had over 51,000 more, with 254,176 ± 4,231 cells (Figure 2A). Note that the strain distribution pattern shows a gradual progression in RBC number from lowest to highest strain, but that the within-strain variation was modest in every strain, the coefficient of variation (CoV) averaging 0.05 across all the strains. From this graded distribution across the strains, coupled with the fact that the RI strains extend beyond the values for the parental strains, one can conclude that there must be multiple genetic variants distinguishing the parental strains that modulate RBC number, and that some of these variant genes work to increase cell number in the presence of the *A* variant while others do so in the presence of the *B* variant, yielding a net additive effect rendering the parental strains nearly identical.

Retinal area is susceptible to variation due to the quality of tissue preservation (Keeley et al., 2017), and within any strain, we observed more substantial variation in average RBC density than we did in estimated total number (average density × retinal area). Because these strains might also differ in the size of their retinas, however, we conducted a linear regression analysis to discern the relationship between total RBC number and retinal area. While these retinas do show some slight modulation in their areal size, there was no significant correlation between RBC number and retinal area (*p* = 0.23; Figure 2B). If, however, we compare the relationship between total RBC number and average RBC density, a strong, significant, correlation is present (*p* = 3.2 × 10^−7^; Figure 2C). Variants discriminating the parental strains must therefore be modulating the size of the RBC population independent of any effect upon retinal area.

### Variation in rod bipolar cell number maps to three genomic loci on Chr 4, 6, and 8

This variation in RBC number across the RI strains mapped multiple QTL (Figure 2D), including three loci with peak LRS scores that reach or surpass the suggestive threshold, on Chr 4 (133.27 Mb; LRS = 14.0), Chr 6 (53.71 Mb; LRS = 10.9), and Chr 8 (98.23 Mb; LRS = 12.7). The locus on Chr 4 showed an effect of *A* alleles correlating with an increase in trait values (19,294 cells), as did the locus on Chr 6 (17,535 cells), while the locus on Chr 8 showed an effect of *B* alleles correlating with an increase in trait values (18,413 cells), their summed magnitude accounting for the entire range of RBC variation across the RI strains. Lesser prospective loci, below the suggestive threshold, were also present (Figure 2D), and composite interval mapping, controlling for the effects of the loci on either Chr 4 or Chr 6, drove two of these lesser loci, on Chr 3 and on Chr 12, respectively, to surpass the suggestive threshold, where the presence of *B* alleles was associated with an increase in trait values at both loci. In sum, this analysis identified multiple prospective genomic loci participating in the control of RBC number, where the countervailing additive effects of *A* vs. *B* alleles render the total number of RBCs in the parental strains to be nearly identical. We restricted our subsequent analysis to the three genomic loci detected on Chrs 4, 6, and 8.

### Bioinformatic analyses identify promising candidate genes at the three QTL

The genomic intervals interrogated at the QTL on Chrs 4, 6, and 8 were 2.1, 5.0, and 7.8 Mb wide, respectively. The interval on Chr 4 contained 55 genes, the interval on Chr 6 contained 70 genes, and interval on Chr 8 contained 96 genes. An extensive bioinformatic analysis was carried out for every gene at these three loci and genes were evaluated if they met two criteria; first, the gene must have allelic variants, either coding or regulatory, that discriminate parental strains from one another. Second, the gene must be expressed in the retina, either during development or in maturity. Of those initial 221 genes, 46 genes on Chr 4, 54 genes on Chr 6, and 85 genes on Chr 8 were confirmed to meet these two criteria (Supplementary Table 2). Genes were further prioritized by the presence of potentially damaging coding variants and/or *cis*-eQTLs and although many genes met these two criteria, one gene at the QTL on Chr 6, *Ggct*, was particularly worthy of further investigation, as explained below.

### *Ggct* is a candidate gene at the QTL on Chr 6

**Figure 3:**
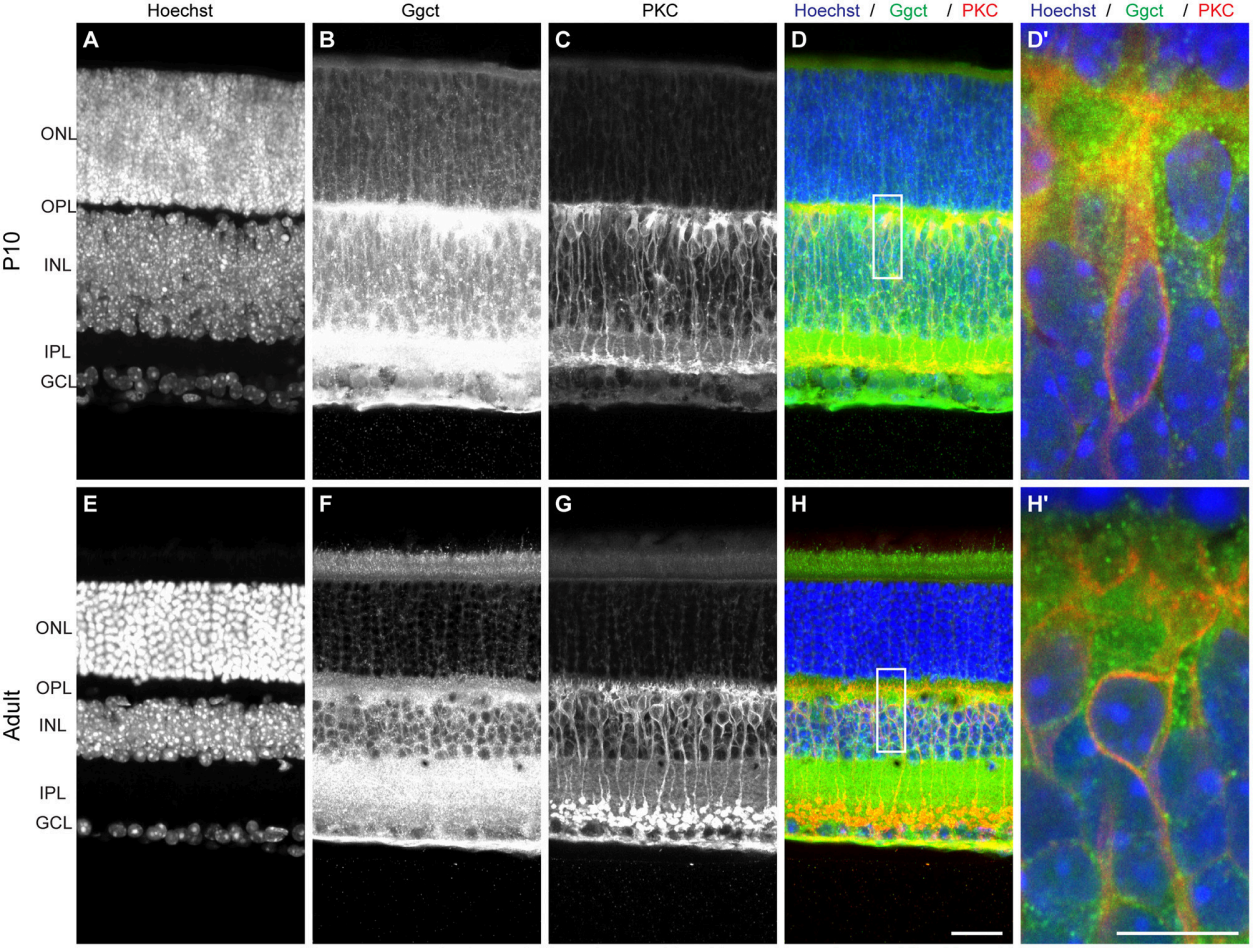
**(A–D)** Ggct immunofluorescence is most conspicuously detected in P10 retinas in the outer portion of the INL and IPL, and is occasionally but not exclusively localized to PKC-positive RBCs. **(E–H)** The same pattern is seen in adult retinas. **(D**′**, H**′**)** Show enlarged regions in **(D,H)**. Scale bar: **(A–H)** = 25 μm; **(D**′**, H**′**)** = 10 μm.

Ggct is an enzyme that plays a role in glutathione degradation (Oakley et al., 2008) and has been shown to be a contributor to a variety of cancers (reviewed in Zhang et al., 2016). Importantly, Ggct has been recently shown to play a role in cell proliferation and death, implicated by *Ggct* knockdown experiments that resulted in an inhibition of proliferation and an induction of apoptosis in human cancer cells (Zhang et al., 2016). Ggct has no known role in retinal development, but it is present in the developing and mature retina where it is most heavily distributed to the IPL and outer portion of the INL, where the RBC population is present (Figure 3). The canonical isoform of the *Ggct* mRNA transcript is 7.8 kb in size, contains 4 exons, and stood out as a candidate gene because it contains a number of variants discriminating the two parental genomes. These include a missense mutation (R45C) in the first exon. In addition, there are 50 SNPs, 14 InDeLs, as well as a single large SV predicted to be 1,075 bp upstream of the TSS (**Figure 6A**).

### *Ggct* expression is negatively correlated with rod bipolar cell number

**Figure 4:**
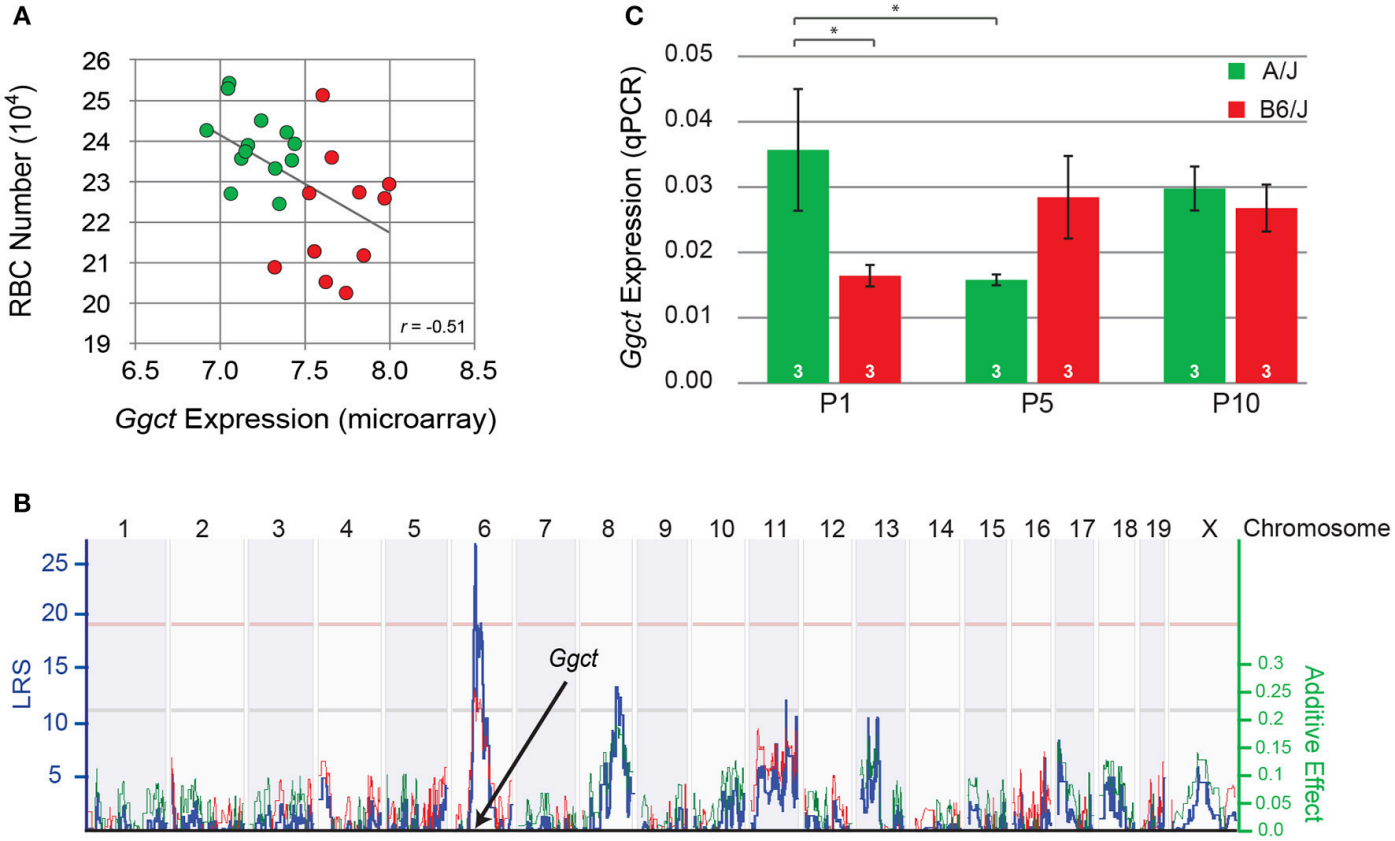
**(A)** Variation in the total number of RBCs was negatively correlated with *Ggct* expression across the RI strain-set, derived from the AXB/BXA RI microarray expression dataset. Those strains carrying the *A* haplotype at the *Ggct* locus are indicated in green, while those carrying the *B* haplotype are shown in red. Each unit on the X axis indicates a doubling in expression. **(B)** Variation in *Ggct* expression mapped a significant *cis*-eQTL at the *Ggct* locus, where the presence of the *B* haplotype (red trace) increased expression. Conventions as in Figure 3. **(C)** qPCR analysis of *Ggct* expression (normalized to housekeeping genes) in the parental strains during the first 10 postnatal days showed a significant interaction, indicating differential expression during the first postnatal week. ^*^p < 0.05.

That a gene-regulatory variant or variants might be playing a role in modulating RBC number across the RI strains comes from a microarray expression database generated from adult whole eye mRNA derived from this same set of strains (Whitney et al., 2011b). *Ggct* expression (probe ILMN 2694767) is observed to exhibit a two-fold variation across the strains, being negatively correlated with the variation in RBC number, with those strains carrying the *B* variant having, as a group, lower numbers of cells (*p* = 0.01; Figure 4A). Note still the continuum of expression across the strains, indicating multiple contributors are regulating *Ggct* expression.

### *Ggct* expression maps a *cis*-eQTL and is differentially regulated during retinal development

The variation in *Ggct* expression across the RI strains itself mapped a significant expression (e)QTL to this same locus on Chr 6 (Figure 4B), indicating some degree of *cis*-regulatory control of transcript levels. Furthermore, *Ggct* expression is increased by the presence of B6/J alleles at this locus. Other suggestive *trans-*eQTLs were also detected on Chrs 8 and 11, consistent with the above observation that multiple independent factors must be modulating transcript levels (Figure 4B). We confirmed expression of *Ggct* during postnatal development in the two parental strains using qPCR, when RBCs are being produced, finding differential expression between them during the first postnatal week (Figure 4C). A two-way ANOVA revealed no main effect of strain (*p* = 0.45) or age (*p* = 0.49), but revealed a significant interaction: in A/J mice, *Ggct* expression was higher at P1 relative to B6/J (*p* = 0.02), and relative to A/J at P5 (*p* = 0.02). In the B6/J strain, conversely, *Ggct* expression appeared to be lower at P1 and then increased by P5, although the corresponding comparisons were not significant (*p* = 0.12), likely due to the higher variability associated with transcript levels at P5 (Figure 4C). *Ggct* is therefore differentially regulated during the period of bipolar cell production, evidenced by the decline in A/J and the suggested increase in B6/J during the first postnatal week. Such dynamic changes in *Ggct* expression may arise from the variable actions of allele-specific *trans-* and *cis-*acting factors as a function of developmental age.

### Electroporation of *Ggct*-encoding plasmids during development reduces bipolar cell number

**Figure 5:**
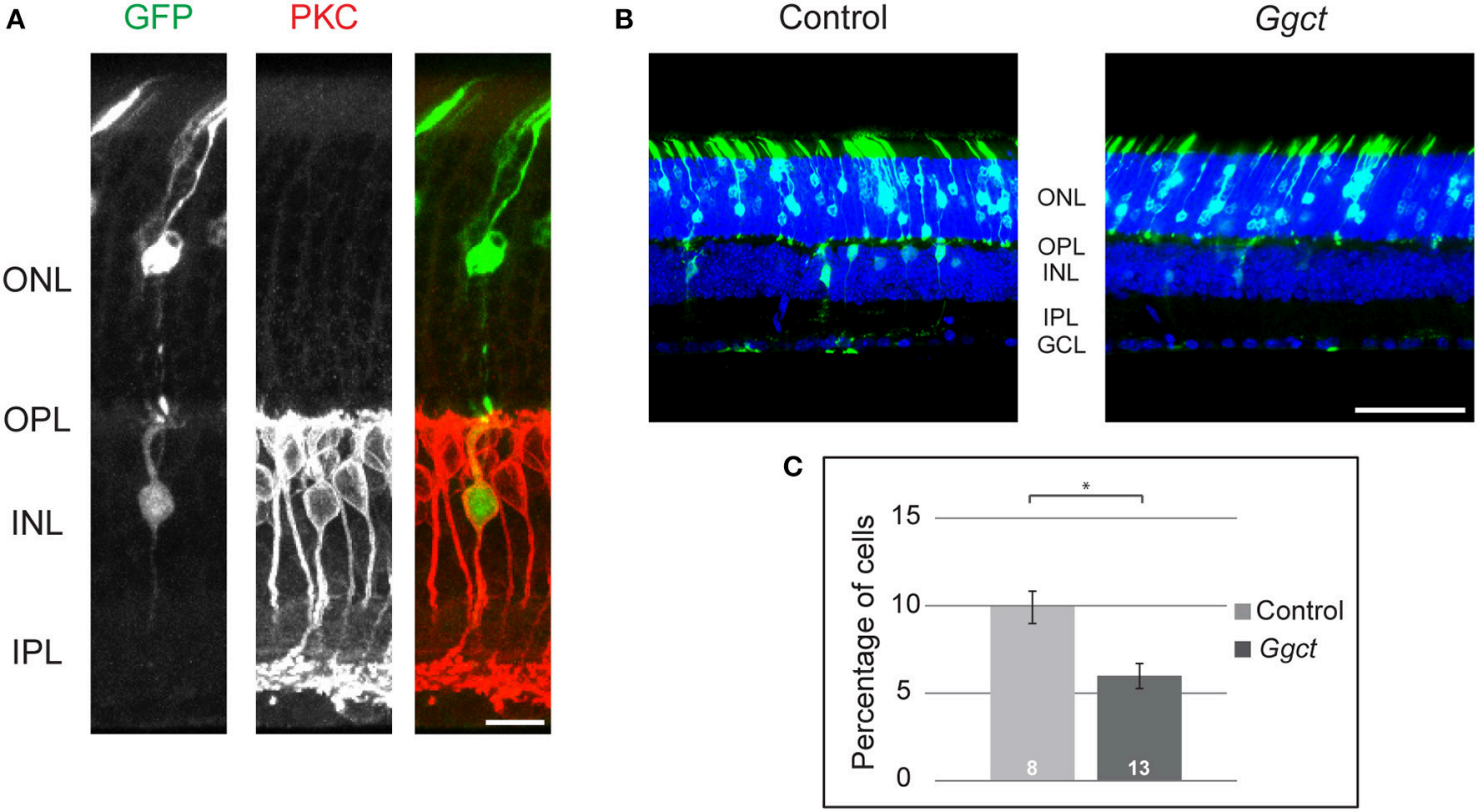
**(A)** Control plasmids electroporated at P2 produced GFP+ cells (green) in the ONL and INL at P21, including some RBCs that were evidenced by their PKC+ status (red). **(B)** *Ggct*-encoding and control plasmids electroporated at P2 produced GFP+ cells in both the ONL and INL at P21. Note the relative dearth of GFP+ cells in the INL relative to the control condition. **(C)** The frequency of GFP+ cells in the outer part of the INL was reduced by nearly 50% following electroporation of *Ggct*-encoding plasmids. *n* = the number of mice analyzed in the two conditions. Scale bar: **(A)** = 10 μm; **(B)** = 50 μm. ^*^p < 0.05.

To examine more directly whether *Ggct* expression levels might play some role in determining RBC number, we expressed *Ggct* using *in vivo* electroporation in one eye of postnatal mice on the day after birth (P2). Plasmids, with or without the *Ggct* coding sequence, were electroporated (*n* = 13 and 8 mice, respectively), and eyes were subsequently examined on P21. An average of 10 sections were quantified from each retina, yielding an average of 955 GFP-positive cells per retina. Initial pilot studies confirmed that a proportion of GFP+ cells were in fact PKC+ (Figure 5A). Because of the low frequency of such double-labeled cells, and due to the inherent variability in electroporation efficiency, we did not label subsequent retinas with antibodies to PKC, instead simply assessing the frequency of GFP+ cells positioned in the outer half of the INL (which should be exclusively bipolar cells) relative to all GFP+ cells found in the section. Figure 5B illustrates representative sections of transfected retinas, showing in the control condition (left) the expected outcome of GFP+ cells found in the ONL and the INL, consistent with the postnatal genesis of rod photoreceptors, bipolar cells, Müller glial cells, and a few amacrine cells (Young, 1985; Matsuda and Cepko, 2004; Morrow et al., 2008; Voinescu et al., 2010). By contrast, Figure 5B (right) reveals the decline in GFP+ cells situated in the bipolar cell division of the INL (the outer INL) when *Ggct* encoding plasmids were electroporated, and this result was borne out by statistical analysis across the two groups of electroporated retinas (Figure 5C): there was a significant reduction in the frequency of GFP+ cells in the outer INL (*p* = 0.01).

These results support the proposed role for *Ggct* in modulating RBC number during the postnatal period. While this analysis did not focus specifically upon the RBCs, the latter are the densest population of bipolar cells in the retina (Wässle et al., 2009; Keeley et al., 2014), and are known to be generated largely after birth (Morrow et al., 2008). Our interpretation of these electroporation studies is consistent with the fact that *Ggct* expression levels across the RI strains correlate inversely with the number of RBCs. That some of the variation in *Ggct* expression across those RI strains maps a *cis*-eQTL encouraged us to examine in further detail the prospective variants expected to modulate expression. As mentioned above, one particularly interesting potential variant was a SV. Structural variants are large and complex DNA arrangements that are thought to account for roughly 1% of all sequence variation in the mouse genome and have been implicated in the pathogenesis of many human diseases (Weischenfeldt et al., 2013).

### A structural variant upstream of *Ggct* discriminates the parental strain genomes

**Figure 6:**
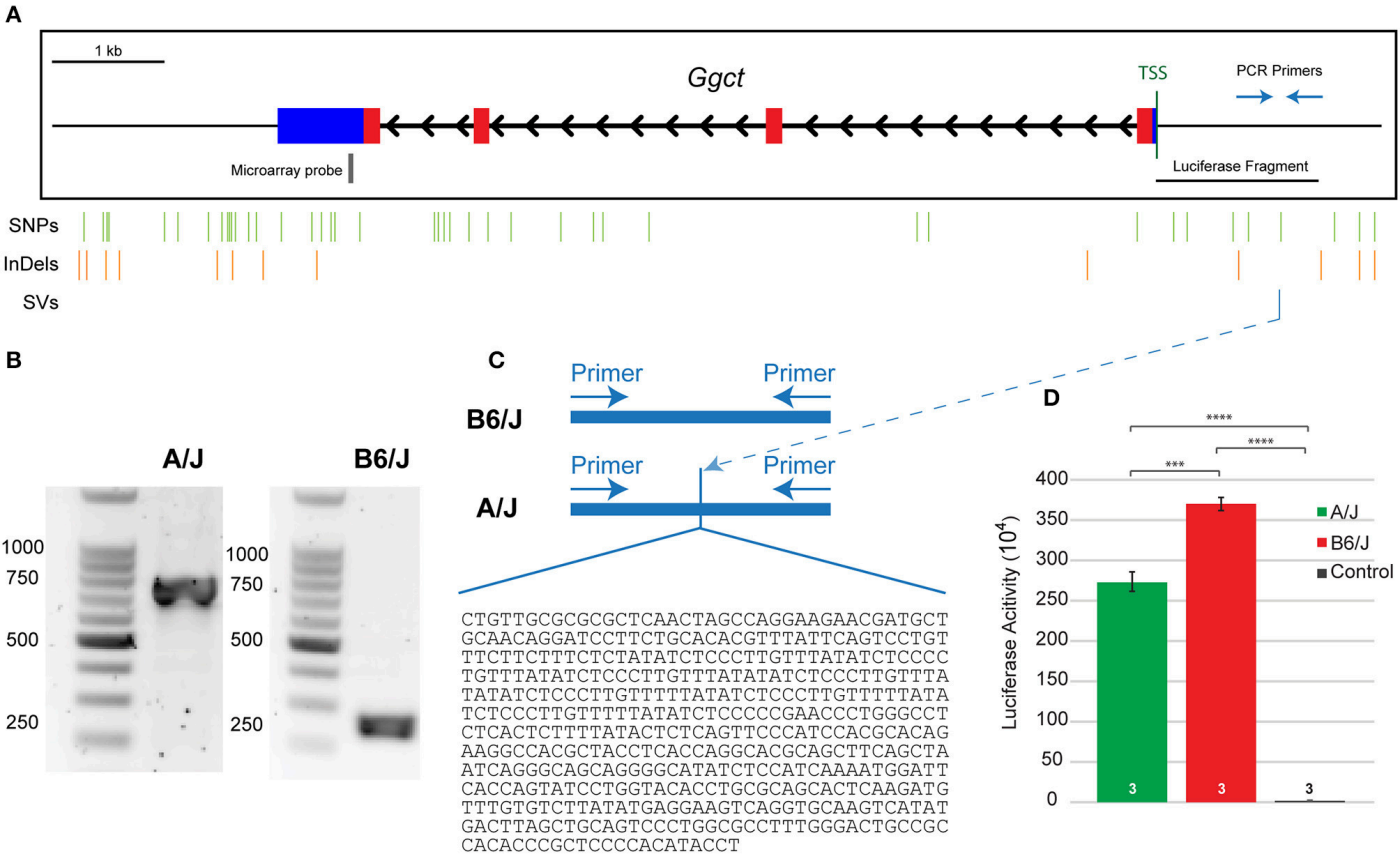
**(A)** The *Ggct* gene contains four exons (colored boxes) and three introns (black arrowheads). Blue boxes represent 5′ and 3′ untranslated regions while thin black lines indicate 2 kb upstream and downstream regions. The position of all single nucleotide polymorphisms (SNPs), short insertions and deletions (InDeLs), and structural variants (SV) that discriminate the parental strains are indicated with vertical lines. Location of PCR primers used in **(C)**, microarray probe used in Figures 4A,B, and the luciferase fragment tested in **(D)** are also shown relative to the TSS. **(B)** The amplified fragment was roughly 500 bp larger in the A/J strain. **(C)** The insertion in A/J was sequenced and confirmed to be 478 bp in size. Nucleotide sequence is listed 5′-3′. **(D)** An *in vitro* luciferase expression assay confirmed that both promoter sequences were effective in driving gene expression, relative to the control condition, but that there was significantly reduced luciferase expression in the presence of the A/J promoter. *n* = the number of biological replicates analyzed per condition. The entire experiment was subsequently replicated twice, obtaining the same significant differences between groups (not shown). ^***^p < 0.001; ^****^p < 0.0001.

According to the Sanger database, the SV upstream of *Ggct* was predicted to begin about 1,075 bp upstream of the TSS. In order to confirm the presence and size of this variant, we amplified this region of the promoter from genomic DNA extracted from A/J and B6/J mice, using primers to known regions surrounding the predicted SV. Figure 6A shows a representation of the *Ggct* gene with the TSS indicated. The 2 kb regions upstream and downstream of the gene are represented, and the location of all variants (SNPs, InDels, and the SV) are shown below. When amplifying this region, we found the structural insertion in the A/J sequence to be approximately 500 bp in size (Figure 6B). We subsequently sequenced this region, determining the insertion in the A/J promoter to be 478 bp (Figure 6C). Given its large size, we hypothesized that this insertion could underlie gene expression changes seen between the two parental strains and sought to test this via luciferase assay.

### The A/J promoter for *Ggct* reduces luciferase expression

We cloned the nucleotide sequences for the B6/J and A/J promoter regions (roughly 1.5 and 2.0 kb fragments, respectively, as the latter included the additional 478 nucleotide sequence insertion—Supplementary Figure 1) into a luciferase vector, and then transfected HEK293T cells with these plasmids to quantify luciferase activity. Both the A/J and B6/J promoters amplify luciferase expression relative to the control plasmid (*p* = 1.3 × 10^−6^ and *p* = 3.7 × 10^−7^ respectively), indicating that both *Ggct* promoter regions are sufficient to drive transcription; there is a significant reduction, however, in luciferase expression when using the A/J promoter relative to the B6/J promoter (*p* = 0.0004; Figure 6D), a finding replicated in two additional independent experiments (data not shown). While we cannot rule out the contribution of other variants in this putative promoter region (Figure 6A), we believe the size of the SV lends itself to being the most likely sequence variant in reducing the transcriptional ability of the A/J *Ggct* promoter; indeed, by identifying potential transcription factor binding sites that differed between the strains, we determined that the SV created 37 unique sites (36 in A/J, 1 in B6/J), while the remaining six variants only created five unique sites (2 in A/J, 3 in B6/J). This suggests that the SV is most likely responsible for the *cis*-eQTL detected in Figure 4B, and for the QTL detected on Chr 6 in Figure 3, and is therefore a quantitative trait variant (QTV).

## Discussion

The present investigation has shown that RBC number is a complex trait controlled by variant genes at multiple genomic loci. B6/J and A/J mice do not vary substantially in RBC number, yet each strain possesses genetic variants that serve to both raise and lower trait values, negating one another's additive contributions to total RBC number. Those RI strains with higher and lower numbers of RBCs possess unique combinations of these variants, defining their total numbers. Indeed, we have identified three large-effect QTLs on Chrs 4, 6, and 8, each of comparable magnitude, though with different valences, such that the presence of the *A* haplotype at the first two loci raise trait values while the *A* haplotype at the third locus reduces trait values. As expected, the two RI strains with the greatest numbers of RBCs possess the *A* haplotype at the loci on Chrs 4 and 6 and the *B* haplotype at the locus on Chr 8, while the five strains with the lowest numbers possess the *B* haplotype at Chrs 4 and 6 loci, and the *A* haplotype at the Chr 8 locus.

Most of the genes that have been identified thus far, participating in the establishment of bipolar cell populations, belong to one of two groups, either basic helix-loop-helix (Bhlh), or homeobox transcription factor families (Burmeister et al., 1996; Tomita et al., 2000; Elshatory et al., 2007; Ohsawa and Kageyama, 2008). Furthermore, only *Bhlhb23* (previously *Bhlhb4*) has been described to have a role in the establishment of RBCs specifically, the most numerous bipolar cell type in the mouse retina (Bramblett et al., 2004; Wässle et al., 2009). The current study uncovers many potential candidate genes that could influence RBC number in the retina; a number of these are being actively pursued, including those on Chrs 4 and 8, but the present investigation focuses attention on one in particular, on Chr 6, being *Ggct*, because of the confluence of evidence suggesting it may play a role in regulating RBC number. *Ggct* does not belong to a transcription factor family, nor has been previously shown to play a role in the developing nervous system; the present results therefore identify a novel genetic contributor in the determination of the RBC population.

*Ggct* was one of a number of candidate genes chosen for further study because its expression correlated with RBC number and it mapped a significant *cis*-eQTL. Ggct was subsequently localized to the bipolar cell stratum of the INL. Furthermore, *Ggct* was differentially expressed during early postnatal development between the two parental mouse strains. These differences between P1 and P5 likely reflect dynamic regulatory control due to one (or more) of the many genetic variants that discriminate the strains. Together these data place *Ggct* in a prime position to influence bipolar cell development via changes in its expression, thereby affecting RBC number in maturity. We subsequently showed that by overexpressing *Ggct* we were able to decrease the number of bipolar cells, as per prediction given that *Ggct* expression is negatively correlated with RBC number. Although this study did not specifically express *Ggct* within RBCs exclusively, it is likely the main contributor to the change in cell number we observe given that RBCs constitute the largest bipolar cell type, and that RBCs are included within the population of transfected cells (e.g., Figure 5A).

*Ggct* has many coding and regulatory variants between the two mouse strains. One of these creates a missense mutation (R45C) within a conserved residue across three different annotated species (human, mouse, and bovine). This amino acid change could potentially be deleterious to protein function given that it changes the side chain from basic (R) to non-basic (C), yet does not fall within a known functional protein domain. While we cannot rule out the consequence of this mutation, we also identified an interesting SV that would feasibly underlie the specific changes in gene expression that we observe. Structural variants are large alterations to DNA sequences that have traditionally been hard to identify until recent advances in sequencing technology. They are also known to be largely pathogenic, associated with specific diseases such as Down syndrome, Angelman syndrome, and Charcot-Marie Tooth disease (Feuk et al., 2006; Weischenfeldt et al., 2013). We consequently examined whether the presence of this large structural insertion was sufficient to alter gene expression. We found that the A/J promoter sequence, including the SV, causes a significant decrease in luciferase activity, relative to the B6/J promoter. The valence of this effect is consistent with our other results, in particular, the negative correlation between *Ggct* expression and RBC number in maturity across the strains, and the decline in bipolar cell number following electroporation of *Ggct*-encoding plasmids. Taken together, these data implicate this SV within the A/J strain as the causal variant responsible for the *cis*-eQTL observed at the locus where *Ggct* resides, ultimately influencing RBC number. While we are unable to dismiss the contribution of other variants within the promoter region, the ~500 bp insertion in the A/J promoter is the most likely contributor to the difference in *Ggct* expression.

The mechanism by which *Ggct* could alter RBC number remains unknown. *Ggct* is upregulated in many human cancers and therefore has been postulated to modulate both apoptotic and cell proliferation pathways (Zhang et al., 2016). For example, when GGCT was silenced in cultured colorectal cancer cells, it was sufficient to trigger caspase-3-mediated apoptosis (Zhang et al., 2016). Furthermore, it is known that decreased levels of intracellular glutathione are associated with cell death and given that *Ggct* is a key player in the glutathione degradation pathway, this further supports the postulation that *Ggct* may be influencing cell number, either directly or indirectly, by modulating the frequency of cell death during development (Cooper and Kristal, 1997). Alternatively, other genes with gamma-glutamyl cyclotransferase activity have been shown to play a role in modulating neurogenesis. For example, Botch has high structural similarity to Ggct, and its enzymatic activity was shown to be critical for antagonizing Notch (Chi et al., 2012, 2014), implicating it in regulating proliferation. Taken together these data provide a framework by which *Ggct* may modulate the specification of cell number within the CNS.

## Author contributions

AK: conducted the candidate gene analysis, Ggct immunofluorescence, qPCR, and luciferase assay; AK and PK: conducted the electroporations and analysis of *Ggct* variants; CA: analyzed electroporated retinas; SB: conducted the RBC counts; PK and BR: conducted the QTL mapping; AK and BR: wrote the manuscript.

### Conflict of interest statement

The authors declare that the research was conducted in the absence of any commercial or financial relationships that could be construed as a potential conflict of interest.
