# Supplementary material for: Genetic Control of Rod Bipolar Cell Number in the Mouse Retina
Source: Front Neurosci. 2018 May 9;12:285. doi: 10.3389/fnins.2018.00285 (PMC5954209; doi:10.3389/fnins.2018.00285)
Supplement: Supplementary file 1 [file Table_1.PDF]

Supplemental Table 1. qPCR primers sequences, product sizes, annealing temperatures, and calculated efficiencies for *Ggct* and 3 housekeeping genes.

Gene	Forward Primer Sequence (5′ to 3′)	Reverse Primer Sequence (5′ to 3′)	Product Size (base pairs)	Annealing Temperature (°C)	Calculated Efficiency
Ggct	CGTTTGCGAA CAGGAGTCTG	GTCTAAGCCCA TCCCCATTCC	130	63	101%
Gapdh	AACTTTGGCA TTGTGGAAGG	GGATGCAGGGA TGATGTTCT	132	60	98%
TBP	CTCAGTTACA GGTGGCAGCA	CAGCACAGAGC AAGCAACTC	120	61.5	94%
β2M	GAGCCCAAGA CCGTCTACTG	GCTATTTCTTT CTGCGTGCAT	134	61.5	97%
