# Supplementary material for: Genetic Control of Rod Bipolar Cell Number in the Mouse Retina
Source: Front Neurosci. 2018 May 9;12:285. doi: 10.3389/fnins.2018.00285 (PMC5954209; doi:10.3389/fnins.2018.00285)
Supplement: Supplementary file 2 [file Table_2.PDF]

Supplemental Table 2: Listed for each of the genes at the QTL on Chrs 4, 6 and 8 are the number of coding variants (STOP gained/lost, frameshift, inframe InDels, initiator codon, splice acceptor/donor/region, and missense); regulatory variants (3’ untranslated region, 5’ untranslated region, 2 kb upstream, 2 kb downstream, and introns); likelihood of missense mutation being damaging to function; presence of significant *cis* -eQTL when mapping variation in gene expression across RI strain-set; and known expression within A/J and B6/J mice from that same AXB/BXA RI expression dataset. Hyphens denote unavailable information. Boxes highlighted in green indicate information of interest, and the corresponding gene to be investigated.

Chromosome 4 QTL

Gene	Genetic Variants											Expression		
	Coding						Regulatory					Damaging Missense	*cis* -eQTL	Adult Expression (Gene Network)
	Stop G/L	Frameshift	Inframe I/D	Initiator	Splice A/D/R	Missense	3' UTR	5' UTR	2kb Upstream	2kb Down-stream	Intron			
Epb4.1	0	0	0	0	0	0	0	0	0	1	1	-	No	-
Snhg12	0	0	0	0	0	0	0	0	0	0	0	-	No	-
Oprd1	0	0	0	0	0	0	0	0	0	0	1	-	No	-
Ythdf2	0	0	0	0	0	0	0	0	1	0	1	-	No	Yes
Gmeb1	0	0	0	0	0	0	0	0	0	1	0	-	No	No?
Taf12	0	0	0	0	0	0	0	0	0	1	0	-	No	Yes
Rab42	0	0	0	0	0	0	0	0	1	0	0	-	No	-
Trspap1	0	0	0	0	0	0	0	0	0	0	0	-	No	-
Rcc1	0	0	0	0	0	0	0	0	2	0	0	-	No	-
Phactr4	0	0	0	0	0	0	1	0	1	1	0	-	No	Yes
4930451E06Rik	0	0	0	0	0	0	0	0	0	0	0	-	No	-
Med18	0	0	0	0	0	0	2	0	5	5	0	-	No	Yes?
Sesn2	0	0	0	0	0	0	0	0	1	3	0	-	No	No?
Atpif1	0	0	0	0	0	0	0	0	2	8	2	-	No	Yes
Dnajc8	0	0	0	0	0	0	1	0	7	12	12	-	No	Yes?
Ptafr	0	0	0	0	0	**1**	14	0	5	31	30	No	No	Yes
Eya3	0	0	0	0	0	0	1	2	112	52	194	-	No	Yes
5830409B07Rik	0	0	0	0	0	0	0	0	0	0	0	-	No	-
Xkr8	0	0	0	0	0	0	8	0	4	4	14	-	**Yes**	Yes
Smpdl3b	0	0	0	0	0	**1**	0	0	0	8	58	No	No	Yes
Rpa2	0	0	0	0	0	0	0	0	2	0	1	-	No	Yes
BC013712	0	0	0	0	0	0	0	0	0	0	0	-	No	-
Ppp1r8	0	0	0	0	0	0	2	0	14	35	16	-	No	Yes
Stx12	0	0	0	0	0	0	2	1	32	21	89	-	No	Yes
C530007A02Rik	0	0	0	0	0	0	0	0	0	0	0	-	No	-
Fam76a	0	0	0	0	0	0	10	0	24	41	130	-	No	Yes
Fgr	0	0	0	0	0	0	0	1	45	1	23	-	No	No
Ahdc1	0	0	**1**	0	0	**1**	10	2	25	124	143	No	No	Yes
Wasf2	0	0	0	0	0	0	21	2	122	180	836	-	No	Yes
Gpr3	0	0	0	0	0	0	6	6	40	87	8	-	No	Yes?
Cd164l2	0	0	0	0	**1**	0	2	2	74	54	77	-	No	No
Map3k6	0	0	0	0	**4**	**6**	1	4	173	105	99	No	**Yes**	Yes
Sytl1	0	0	**1**	0	**2**	**6**	1	0	30	0	131	No	No	Yes
Tmem222	0	0	0	0	0	0	1	1	107	68	102	-	No	Yes
Wdtc1	0	0	0	0	**3**	0	16	2	174	212	732	-	No	No
NHE1 (Slc9a1)	0	0	0	0	0	0	16	0	0	0	214	-	No	Yes
1700091J24Rik	0	0	0	0	0	0	0	0	0	0	0	-	No	-
Fam46b	0	0	0	0	**1**	**3**	5	0	37	38	35	Yes	No	-
Trnp1	0	0	0	0	0	0	8	1	53	9	47	-	No	Yes
Kdf1	0	0	0	0	0	**1**	2	2	137	71	156	No	No	Yes
Nudc	0	0	0	0	0	0	2	0	163	14	192	-	No	Yes
Nr0b2	0	0	0	0	0	0	0	0	88	54	8	-	No	No
Gpatch3	0	0	**1**	0	**2**	**5**	5	0	112	65	133	-	No	No
Gpn2	0	0	0	0	**1**	0	8	2	73	147	81	Yes	No	-
Sfn	0	0	0	0	0	0	3	0	22	22	0	-	No	No?
Zdhhc18	0	0	0	0	0	0	22	0	167	200	568	-	No	Yes?
4930429E23Rik	0	0	0	0	0	0	0	0	0	0	0	-	No	-
Pigv	0	0	0	0	0	0	13	0	22	78	95	-	No	No

1700041L08Rik	0	0	0	0	0	0	0	0	0	0	0	-	No	-
Arid1a	0	0	**2**	0	**1**	0	0	5	87	57	368	-	No	No?
Aim1l	0	0	0	0	0	0	0	0	0	0	0	-	No	-
Rps6ka1	0	0	0	0	**2**	0	10	7	146	200	360	-	No	Yes
Hmgn2	0	0	0	0	**1**	0	3	4	43	70	19	-	**Yes**	Yes
Dhdds	0	0	0	0	**1**	0	7	3	157	109	284	-	**Yes**	Yes
Lin28a	0	0	0	0	0	0	14	2	90	95	145	-	No	No

Chromosome 6 QTL														
Gene	Genetic Variants											Expression		
	Coding						Regulatory					Damaging Missense	cis-eQTL	Adult Expression (Gene Network)
	Stop G/L	Frameshift	Inframe I/D	Initiator	Splice A/D/R	Missense	3' UTR	5' UTR	2kb Upstream	2kb Down-stream	Intron			
Hoxa1	0	0	**1**	0	0	0	0	0	0	5	0	-	No	-
Hoxa2	0	0	0	0	0	0	1	0	0	1	0	-	No	No
Hoxaas2	0	0	0	0	0	0	0	0	0	0	0	-	No	No
Hoxa3	0	0	0	0	0	0	1	0	14	4	5	-	No	-
5730596B20Rik	0	0	0	0	0	0	0	0	9	8	0	-	No	No
Hoxa4	0	0	0	0	0	**1**	1	7	5	9	0	**Yes**	No	No
Hoxaas3	0	0	0	0	0	0	0	0	0	0	0	-	No	No
Hoxa5	0	0	0	0	0	0	0	0	1	0	0	-	No	-
Hoxa6	0	0	0	0	0	0	0	0	0	0	0	-	No	No
Hoxa7	0	0	0	0	0	0	0	0	0	0	0	-	No	-
Hoxa9	0	0	0	0	0	0	0	0	1	0	2	-	No	-
9930038K12Rik	0	0	0	0	0	0	0	0	0	0	0	-	No	No
Hoxa10	0	0	0	0	0	0	0	0	2	1	3	-	No	
Hoxa11	0	0	0	0	0	0	0	0	0	2	0	-	No	No
Hoxa11os	0	0	0	0	0	0	0	0	0	0	0	-	No	No
9530018H14Rik	0	0	0	0	0	0	0	0	0	0	0	-	No	-
Hoxa13	0	**2**	0	0	**2**	**2**	0	4	0	0	5	No	No	-
Evx1os	0	0	0	0	0	0	0	1	1	0	0	-	No	No?
Evx1	0	0	0	0	0	0	0	0	0	2	0	-	No	-
1700094M24Rik	0	0	0	0	0	0	0	0	0	0	0	-	No	Yes
Hibadh	0	0	0	0	0	0	0	0	0	1	18	-	No	-
Tax1bp1	0	0	0	0	0	0	0	0	5	9	15	-	No	Yes
Jazf1	0	0	0	0	0	0	0	0	3	0	48	-	No	Yes
Gm4872	0	0	0	0	0	0	0	0	0	0	0	-	No	Yes?
Creb5	0	0	0	0	0	**1**	4	0	16	8	451	No	**Yes**	-
Tril	0	0	0	0	0	0	1	0	1	2	0	-	No	Yes?
Gm16499	0	0	0	0	0	0	0	0	2	1	0	-	No	Yes
Cpvl	0	0	0	0	0	0	0	0	41	1	47	-	No	-
4921529L05Rik	0	0	0	0	0	0	0	0	0	0	0	-	No	No
4930590G02Rik	0	0	0	0	0	0	0	0	0	0	0	-	No	-
9530036M11Rik	0	0	0	0	0	0	0	0	0	0	0	-	No	-
Chn2	0	0	0	0	0	**2**	3	0	80	97	940	No	No	-
9030405F24Rik	0	0	0	0	0	0	0	0	0	0	0	-	No	No
Prr15	0	0	0	0	0	0	0	6	71	58	0	-	No	-
9130019P16Rik	0	0	0	0	**1**	0	0	0	3	13	840	-	No	Yes
Wipf3	0	0	0	0	0	0	0	1	2	3	17	-	No	No
Scrn1	0	0	0	0	0	0	1	2	77	71	268	-	No	Yes
Fkbp14	0	0	0	0	0	0	0	0	15	3	4	-	No	Yes
Plekha8	0	0	0	0	0	0	0	1	0	2	19	-	No	Yes
Mturn	0	0	0	0	0	0	8	0	24	27	56	-	No	Yes
2610209C05Rik	0	0	0	0	0	0	0	0	0	0	0	-	No	Yes
Znrf2	0	0	0	0	0	0	1	0	1	0	9	-	No	-
Nod1	0	0	0	0	0	0	0	1	5	2	69	-	No	Yes
Ggct	0	0	0	0	0	**1**	52	0	36	81	16	**Yes**	**Yes**	Yes
Gars	0	0	0	0	0	**1**	0	0	3	9	120	No	No	Yes
Crhr2	0	0	0	0	0	**1**	3	8	16	26	34	No	No	Yes
Inmt	0	0	0	0	0	0	1	0	71	61	53	-	**Yes**	Yes?
Fam188b	0	0	0	0	0	**1**	1	1	21	2	387	**Yes**	No	Yes

Aqp1	0	0	0	0	0	0	1	0	2	7	3	-	No	No
**Ghrhr**	0	0	0	0	**1**	0	0	0	30	9	55	-	No	Yes
6430584L05	0	0	0	0	0	0	0	0	0	0	0	-	No	No
Adcyap1r1	0	0	0	0	0	0	11	2	40	51	101	-	No	-
Neurod6	0	0	0	0	0	0	0	0	0	0	0	-	No	No
Ccdc129	0	0	0	0	0	0	0	0	1	0	3	-	No	-
Gsbs	0	0	0	0	0	0	0	0	0	0	0	-	No	Yes
Pde1c	0	0	0	0	0	0	0	0	1	1	11	-	No	-
Lsm5	0	0	0	0	0	0	0	0	0	0	0	-	No	Yes?
4933427C19Rik	0	0	0	0	0	0	0	0	0	0	0	-	No	-
Avl9	0	0	0	0	0	0	0	0	0	0	0	-	No	-
3110035G12Rik	0	0	0	0	0	0	0	0	0	0	0	-	No	-
Kbtbd2	0	0	0	0	0	0	0	0	0	0	0	-	No	-
Fkbp9	0	0	0	0	0	0	0	0	0	0	0	-	No	-
Nt5c3	0	0	0	0	0	0	0	0	0	0	0	-	No	-
Vmn1r4	0	0	0	0	0	0	0	0	0	0	3	-	No	-
Vmn1r5	0	0	0	0	0	0	0	0	0	0	0	-	No	-
Vmn1r6	0	0	0	0	0	0	0	0	0	0	0	-	No	-
Vmn1r8	0	0	0	0	0	0	0	0	0	0	0	-	No	-
Vmn1r9	0	0	0	0	0	0	0	0	0	0	0	-	No	-
Vmn1r10	0	0	0	0	0	0	0	0	1	0	0	-	No	-
Vmn1r11	0	0	0	0	0	0	0	0	0	0	0	-	No	No

Chromosome 8 QTL

Gene	Genetic Variants											Expression		
	Coding						Regulatory					Damaging Missense	cis-eQTL	Adult Expression (Gene Network)
	Stop G/L	Frameshift	Inframe I/D	Initiator	Splice A/D/R	Missense	3' UTR	5' UTR	2kb Upstream	2kb Down-stream	Intron			
Gm2716	0	0	0	0	0	0	0	0	0	0	0	-	No	-
**Tmem188**	0	0	0	0	**2**	0	19	0	55	37	145	-	No	Yes?
Heatr	0	0	0	0	0	**1**	15	4	158	93	269	No	No	-
9430002A10Rik	0	**1**	0	0	0	**3**	0	0	46	116	0	No	No	No
Adcy7	0	0	0	0	**2**	0	0	5	254	0	290	-	No	No?
Brd7	0	0	0	0	0	0	0	0	0	0	0	-	No	Yes
Nkd1	0	0	0	0	0	0	0	0	0	0	1	-	No	Yes
Papd5	0	0	0	0	0	0	3	2	102	86	481	-	No	No
Snx20	0	0	0	0	0	0	0	0	0	0	0	-	No	No
Nod2	0	0	0	0	0	0	0	0	0	1	1	-	No	No
Cyld	0	0	0	0	0	0	0	0	0	0	0	-	No	Yes?
Sall1	0	0	0	0	0	0	0	0	0	0	0	-	No	Yes
Rps6-ps2	0	0	0	0	0	0	0	0	0	0	0	-	No	-
Tox3	0	0	0	0	0	0	0	0	0	0	1	-	No	Yes
4930405E02Rik	0	0	0	0	0	0	0	0	0	0	0	-	No	-
Chd9	0	0	0	0	0	0	0	0	0	0	13	-	No	Yes
EG626231	0	0	0	0	0	0	0	0	0	0	0	-	No	-
Rbl2	0	0	0	0	0	0	0	0	0	0	0	-	No	No
Aktip	0	0	0	0	0	0	0	0	0	1	1	-	No	Yes
Rpgrip1l	0	0	0	0	0	0	0	0	0	0	1	-	No	-
**Fto**	0	0	0	0	0	**1**	0	0	0	5	11	No	No	Yes?
B130011D17Rik	0	0	0	0	0	0	0	0	0	0	0	-	No	-
4831440D22Rik	0	0	0	0	0	0	0	0	0	0	0	-	No	-
Irx3	0	0	0	0	0	0	0	0	0	4	0	-	No	Yes
Irx3os	0	0	0	0	0	0	0	0	0	0	0	-	No	No
Crnde	0	0	0	0	0	0	0	0	0	3	2	-	No	Yes
Irx5	0	0	0	0	0	0	0	0	0	0	0	-	No	Yes
Irx6	0	0	0	0	0	0	0	0	0	0	0	-	No	Yes
**Mmp2**	0	0	0	0	**4**	0	3	1	24	33	315	-	No	Yes
**Lpcat2**	0	0	0	0	**1**	**5**	13	0	82	160	978	Yes	**Yes**	Yes
**Capns2**	0	0	0	0	0	**1**	0	2	82	42	4	Yes	No	Yes
**Slc6a2**	0	0	0	0	**2**	0	44	3	66	58	492	-	No	No
**Ces1a**	0	0	0	0	**3**	**6**	3	0	72	70	411	No	No	-

**Ces1b**	0	**1**	0	0	**8**	**11**	0	2	29	23	426	-	No	-
**Ces1c**	0	0	0	0	**1**	**13**	6	0	192	166	484	-	No	-
Ces1d	0	0	0	0	0	0	0	0	0	0	1	-	No	-
Ces1e	0	0	0	0	0	0	0	0	0	0	0	-	No	-
Ces1f	0	0	0	0	0	0	0	0	1	0	1	-	No	-
Ces1g	0	0	0	0	0	0	0	0	0	0	1	-	No	-
Ces5a	0	0	0	0	0	0	0	0	0	0	2	-	No	-
Gnao1	0	0	0	0	0	0	1	0	0	0	7	-	No	Yes?
4930488L21Rik	0	0	0	0	0	0	0	0	0	0	0	-	No	-
Amfr	0	0	0	0	0	0	0	0	0	1	0	-	No	Yes?
Nudt21	0	0	0	0	0	0	0	0	0	0	0	-	No	Yes
Ogfod1	0	0	0	0	0	0	0	0	0	0	1	-	No	Yes
Bbs2	0	0	0	0	0	0	0	0	0	1	0	-	No	Yes
Mt4	0	0	0	0	0	0	0	0	0	0	0	-	No	Yes
Mt3	0	0	0	0	0	0	0	0	0	0	0	-	No	Yes
Mt2	0	0	0	0	0	0	0	0	0	0	0	-	No	Yes
Nup93	0	0	0	0	0	0	0	0	0	0	1	-	No	Yes?
**Slc12a3**	0	0	0	0	0	0	0	0	0	1	1	-	**Yes**	Yes?
Herpud1	0	0	0	0	0	0	0	0	9	21	17	-	No	Yes
9330175E14Rik	0	0	0	0	0	0	0	0	0	0		-	No	No
**Nrlc5**	0	0	0	0	**2**	**10**	3	0	10	0	181	Yes	No	-
Tmem28	0	0	0	0	0	0	0	0	0	0		-	No	No
Cpne2	0	0	0	0	0	0	0	0	1	11	28	-	No	Yes
Fam192a	0	0	0	0	0	0	0	1	1		7	-	No	-
Rspry1	0	0	0	0	0	0	0	0	4	4	13	-	No	Yes
**Arl2bp**	0	0	0	0	0	0	0	0	3	0	1	-	**Yes**	Yes
Pllp	0	0	0	0	0	0	0	0	0	0	3	-	No	Yes
Ccl22	0	0	0	0	0	0	0	0	1	1	0	-	No	No?
Cx3cl1	0	0	0	0	0	0	2	0	8	28	12	-	No	Yes?
1700121C10Rik	0	0	0	0	0	0	0	0	0	0	0	-	No	-
Ccl17	0	0	0	0	0	0	1	0	17	9	2	-	No	Yes
**Ciapin1**	0	0	0	0	**2**	0	14	8	209	87	139	-	No	Yes
**Coq9**	0	0	0	0	**2**	0	8	0	162	157	246	-	No	Yes
Polr2c	0	0	0	0	0	0	0	0	34	6	0	-	No	No
Dok4	0	0	0	0	0	0	1	1	22	6	18	-	No	Yes
Ccdc102a	0	0	0	0	0	0	0	0	40	30	26	-	No	Yes?
**Gpr114**	0	0	0	0	0	**1**	10	0	39	48	138	Yes	No	No
ENSMUSG00000070	0	0	0	0	0	0	0	0	0	0	0	-	No	-
Gm10286	0	0	0	0	0	0	0	0	7	7	0	-	No	-
**Gpr56**	0	0	0	0	0	**2**	13	3	76	38	254	No	No	No
**Gpr97**	0	0	0	0	**1**	**4**	9	0	48	34	258	No	No	No
**Ccdc135**	0	0	0	0	**1**	0	4	0	19	15	41	-	No	Yes
Katnb1	0	0	0	0	0	0	2	0	31	7	26	-	No	Yes?
**Kifc3**	0	0	0	0	0	**3**	1	3	53	2	81	No	**Yes**	Yes
**Cngb1**	0	0	0	0	**2**	**2**	0	1	0	0	0	No	No	Yes
**Cngb1**	0	0	**15**	0	**3**	**1**	1	1	0	0	202	-	No	Yes
Tepp	0	0	0	0	0	0	0	2	44	85	100	-	No	-
Zfp319	0	0	0	0	0	0	0	0	4	20	0	-	No	No
**Usb1**	0	0	0	0	**1**	**1**	2	2	55	67	79	No	No	-
**Mmp15**	0	0	0	0	0	0	4	0	30	19	52	-	**Yes**	Yes
Gtl3	0	0	0	0	0	0	0	0	3	0	8	-	No	Yes
Csnk2a2	0	0	0	0	0	0	4	0	1	15	95	-	No	Yes
4933406B17Rik	0	0	0	0	0	0	0	0	0	0	0	-	No	No
Ccdc113	0	0	0	0	0	0	0	0	5	48	46	-	No	Yes
1700112L15Rik	0	0	0	0	0	0	0	0	0	0	0	-	No	-
**Prss54**	0	0	0	0	0	**1**	0	0	30	10	204	Yes	No	-
Gins3	0	0	0	0	0	0	0	0	7	1	3	-	No	Yes
Ndrg4	0	0	0	0	0	0	2	2	60	16	91	-	No	Yes
Setd6	0	0	0	0	0	0	0	0	17	13	8	-	No	Yes?
**Cnot1**	0	0	0	0	**1**	0	2	0	0	12	41	-	No	No?
4930513N10Rik	0	0	0	0	0	0	0	0	1	1	2	-	No	No
Slc38a7	0	0	0	0	0	0	1	0	1	4	1	-	No	Yes
Got2	0	0	0	0	0	0	1	0	0	3	8	-	No	Yes
