# Supplementary material for: Genetic Control of Rod Bipolar Cell Number in the Mouse Retina
Source: Front Neurosci. 2018 May 9;12:285. doi: 10.3389/fnins.2018.00285 (PMC5954209; doi:10.3389/fnins.2018.00285)
Supplement: Supplementary file 3 [file Image_1.PDF]

Supplemental Figure 1. Promoter sequences used in the *in vitro* luciferase expression assay. The insertion in the A/J strain is highlighted in yellow.

A/J Promoter Sequence

(-1890)GAACTCACAGAGAACTGCCTGCCTCTGCCTCTCGAGTGATGGTATTAAAGGTGC
AGTCCACAATGCCCAGCTTCATGTTGCTTTCTGACTGTGTCAGGATAGTCTTCTTTTATTG
GTGACCAGATTGACTGGCTATCAGGTGAGAGAGGTCAGGTTTCCCAAATAATCATGGTCTT
CCTGTCCAATGACTAGCAGTGAGCCTTGGGGGATAACTGAGACTCTATAAACATCCCTGTG
ACCTGATGCTCCTGACGGGAAGTTTGGTTGCACCGGAGACAGATGTTCTAATCCCAGTACC
CCCATTTCCTGCATTTAGCCAGTATACCTCTGTTGCGCGCGCTCAACTAGCCAGGAAGAAC
GATGCTGCAACAGGATCCTTCTGCACACGTTTATTCAGTCCTGTTTCTTCTTTCTCTATAT
CTCCCTTGTTTATATCTCCCCTGTTTATATCTCCCTTGTTTATATATCTCCCTTGTTTATA
TATCTCCCTTGTTTTTATATCTCCCTTGTTTTTATATCTCCCTTGTTTTTATATCTCCCCC
GAACCCTGGGCCTCTCACTCTTTTATACTCTCAGTTCCCATCCACGCACAGAAGGCCACGC
TACCTCACCAGGCACGCAGCTTCAGCTAATCAGGGCAGCAGGGGCATATCTCCATCAAAAT
GGATTCACCAGTATCCTGGTACACCTGCGCAGCACTCAAGATGTTTGTGTCTTATATGAGG
AAGTCAGGTGCAAGTCATATGACTTAGCTGCAGTCCCTGGCGCCTTTGGGACTGCCGCCAC
ACCCGCTCCCCACATACCTTTTTTTAAAAAAGCAAAGAATTGCTTCCTCATCAGCTATTTG
GTTTTCAAAAATACAGTTGGTACTAGAAAGGCAGAACACTCATTTTCTTAAATACCCACTT
TTTAAAAGTGATGAAGTGTTAGCCACTGATGGCATGGCAATATGGTTCTCTTCTCTGTTTT
AATTTGGTTTACTTCCTGAAGGGAATGACATACATTTAGGGATCATTTTAGAGTTTCCAGT
CGTTCTGCTCCTTAAGTTGTTCTCCCTTTGTCTAGTGAATGGTCTTTTAGGAATGACCTTG
TTCATCTTGGGGTTCTCATTTTCTGGATTATTGTTCTTTCAAGAGGTATTTTCACTCCCAG
CTTTGACCTAAGATTCAGTCATTTCTCCAGGAATCCTGGCACCTGTCAGTGTGGAGTTGAA
TTTAGATTAATTTCTGAGGGCCAACGGCAGTCATTGCTCTAGACCTTCAGTTTGCTCTCCT
ATCTTCATTTCAAGAGCAACAACATGGGTGTTTTGTGTCGTTCTATTTTAAAAAGAGGAAA
TAGATTGCAACGATGAACAGAGTCAACTGGCTAGCAAATGGCATGTCCGGATCTGAACGTG
GGAGAAAGACTACCCTGGTATGCAGTGAGGCAACTTCAGGTAGTGATCCTAAGAAAACTTG
TAGGATCAGCCTTCCCATTCCAGGAAGGCTGAGGCAGGAGAACTGCCGCAAGATCCAGGCT
AGGCCGACAGCAATTTGAGACTTGTCTCCTCTATACCAGGCGCAGAGCCTACCGCACCACT
GGTTGACCTGGGCAATCGCCACCAGGTGGGGAAAACACAGTCGCCAGTAGCCACTCTCGGG
CTGTCCGTTAGACTCGCCAATCAGCGGCGCCTCAGGCAGGGGGCCGTACATCCGAGGGATG
CATCCAATAGAAAAGCGACGCCAGTGGGCGGGAGGAGCTGTAGACAGGCCAATCCTGGTAG
TTAGCTCACGCCGGCAAGCCGGCAGCCAATCCTCAGCGCGCTCTCTGTGCGGCTCCGCCCG
CCGCTCTGCACTACCGCGTGGGTGGCGGCGCACTGCTCTTTTTAGCAGCGCTAGTCCGGCT
TCTCTG(-1)

B6/J Promoter Sequence

(-1413)GAACTCACAGAGAACTGCCTGCCTCTGCCTCTCGAGTGATGGTATTAAAGGTGC
AGTCCACAATGCCCAGCTTCATGTTGCTTTCTGACTGTGTCAGGATAGTCTTCTTTTATTG
GTGACCAGATTGACTGGCTATCAGGTGAGAGAGGTCAGGTTTCCCAAATAATCATGGTCTT
CCTGTCCAATGACTAGCAGTGAGCCTTGGGGGATAACTGAGACTCTATAAACATCCCTGTG
ACCTGATGCTCCTGACGGGAAGTTTGGTTGCACCAGAGACAGATGTTCTAATCCCAGTACC
CCCATTTCCTGCATTTAGCCAGTATACTTTTTTTAAAAAAGCAAAGAATTGCTTCCTCAT
CAGCTATTTGGTTTTCAAAAATACAGTTGGTACTAGAAAGGCAGAACACTCATTTTCTTAA
ATACCCACTTTTTAAAAGTGATGAAGTGTTAGCCACTGATGGCATGGCAATATGGTTCTCT
TCTCTGTTTTAATTTGGTTTACTTCCTGAAGGGAATGACATACATTTAGGGATCATTTTAG
AGTTTCCAGTCGTTCTGCTCCTTAAGTTGTTCTCGCTTTGTCTAGTGAATGGTCTTTTAGG
AATGACCTTGTTCATCTTGGGGTTCTCATTTTCTGGATTATTGTTCTTTCAAGAGGTAATT
TTCACTCCCAGCTTTGACCTAAGATTCAGTCATTTCTCCAGGAATTCTGGCACCTGTCAGT
GTGGAGTTGAATTTAGATTAATTTCTGAGGGCCAACGGCAGTCATTGCTCTAGACCTTCAG
TTTGCTCTCCTATCTTCATTTCAAGAGCAACAACATGGGTGTTTTGTGTCGTTCTATTTTA
AAAAGAGGAAATAGATTGCAACGATGAACAGAGTCAACTGGCTAGCAAATGGCATGTCCGG
ATCTGAACGTGGGAGAAAGACTACCCTGGTATGCAGTGAGGCAACTTCAGGTAGTGATCCT
AAGAAAACTTGTAGGATCAGCCTTCCCATTCCAGGAAGGCTGAGGCAGGAGAACTGCCGCA
AGATCCAGGCTAGGCCGACAGCAATTTGAGACTTGTCTCCTCTATACCAGGCGCAGAGCCT
ACCGCACCACTGGTTGACCTGGGCAATCGCCGCCAGGTGGGGAAAACACAGTCGCCAGTAG
CCACTCTCGGGCTGTCCGTTAGACTCGCCAATCAGCGGCGCCTCAGGCAGGGGGCCGTACA
TCCGAGGGATGCATCCAATAGAAAAGCGACGTCAGTGGGCGGGAGGAGCTGTAGACAGGCC
AATCCTGGTAGTTAGCTCACGCCGGCAAGCCGGCAGCCAATCCTCAGCGCGCTCTCTGTGC
GGCTCCGCCCGCCGCTCTGCACTACCGCGTGGGTGGCGGCGCACTGCTCTTTTTAGCAGCG
CTAGTCCGGCTTCTCTG(-1)
